# Females with paired occurrence of cancers in the UADT and genital region have a higher frequency of either Glutathione S-transferase *M1/T1 *null genotype

**DOI:** 10.1186/1477-3163-4-6

**Published:** 2005-03-24

**Authors:** Sameer G Jhavar, Rajiv Sarin, Supriya Chopra, Ashwin Kotnis, Rita Mulherkar, Roger A'Hern, Jai Prakash Agarwal, Shyam Kishore Shrivastava, Ketayun A Dinshaw

**Affiliations:** 1 Departments of Radiation Oncology, Tata Memorial Centre, Mumbai, India; 2 Cancer genetics Unit, Tata Memorial Centre, Mumbai, India; 3 Section of Genetic Engineering, Tata Memorial Centre, Mumbai, India; 4 Royal Marsden Hospital NHS Trust, London, UK

**Keywords:** Glutathione S-transferase, second cancer, paired cancer, multiple cancer, squamous carcinoma, genital, UADT

## Abstract

Upper Aero digestive Tract (UADT) is the commonest site for the development of second cancer in females after primary cervical cancer. Glutathione S-transferase (*GSTM1 *and / or *T1*) null genotype modulates the risk of developing UADT cancer (primary as well as second cancer). The aim of this study was to evaluate the difference in *GST *null genotype frequencies in females with paired cancers in the UADT and genital region as compared to females with paired cancers in the UADT and non-genital region. Forty-nine females with a cancer in the UADT and another cancer (at all sites-genital and non-genital) were identified from a database of patients with multiple primary neoplasms and were analyzed for the *GSTM1 *and *T1 *genotype in addition to known factors such as age, tobacco habits, alcohol habits and family history of cancer. Frequencies of *GSTM1 *null, *GSTT1 *null, and *either GSTM1/T1 *null were higher in females with paired occurrence of cancer in the UADT and genital site (54%, 33% and 75% respectively) in comparison to females with paired occurrence of cancer in the UADT and non-genital sites (22%, 6% and 24% respectively). The significantly higher inherited frequency of either *GSTM1*/*T1 *null genotype in females with a paired occurrence of cancers in UADT and genital region (p = 0.01), suggests that these females are more susceptible to damage by carcinogens as compared to females who have UADT cancers in association with cancers at non-genital sites.

## Introduction

Precise risk estimation for the development of second cancers in the Head & Neck, Lung, and Esophagus, also known as the Upper Aero-Digestive Tract (UADT), is difficult due to the multifactorial origin and gene-environment interaction [1].

The Glutathione S transferase (*GST*) family of enzymes coded for by at least five distinct loci-*alpha*, *mu*, *theta*, *pi *and *gamma *detoxify tobacco carcinogens [2] and play an important role in the gene-environment interaction associated risk for cancer. The results of a recently published meta- and pooled- analysis of 4635 cases and 5770 controls, support the widely studied association of inherited *GST *genotype with the risk of developing squamous cell carcinomas (SCC) in the UADT. In addition the results support the notion that the risk increases when genotypes at multiple *GST *loci are considered [3]. Patients with second (synchronous and metachronous) cancers in the UADT also have a higher frequency of *GST *null genotype (particularly *GSTM1 *and *T1*) as compared to those with single cancers [4-7].

Similar to the association seen in UADT cancers, *GST *null genotype has been associated with the risk of developing Human Papilloma Virus (HPV)-positive primary cervical cancer in females [8,9] and also with other cancers such as primary breast cancer [10] and primary thyroid cancer [11].

Females with a primary HPV related cancer (particularly uterine cervix) develop second cancers in the UADT with an increased frequency [12-14]. HPV transmission has been suggested to account in part for the paired occurrence of cancers at these anatomically distinct sites of UADT and cervix. Exposure to tobacco carcinogens has been suggested as synergistic cofactor [15,16]. The mechanism by which HPV infection, necessary for the development of cervical cancer [17], causes UADT cancers is unclear [18]. Recently it has been shown that interaction between viral infection (especially HPV) and xenobiotic enzymes such as *GSTM1 *could modulate cancer risk, however the evidence comes from *in vitro *studies alone [19].

There are no studies found in the literature evaluating the association of *GST *null genotypes and the risk of developing second cancers in the UADT in females, particularly after primary cancers at genital sites. We identified females with a paired cancer in the UADT in association with another cancer (at all sites-genital and non-genital) and analyzed *GSTM1 *and *T1 *genotype in addition to known factors such as age, tobacco habits, alcohol habits and family history of cancer.

## Patients and methods

### Patients

From a registry of 150 patients with Multiple Primary Neoplasms (MPN) at Tata Memorial Hospital, Mumbai, during the period of 1996 – 2004, all the females, who met the following criteria were selected: 1) synchronous or metachronous occurrence of second primary cancer; 2) Histological or cytological confirmation of both primaries; 3) One of the two cancers was a SCC in the UADT. Modified Hong's criteria [20] was used to differentiate the second primary cancer from metastatic or direct spread from the first cancer.

Details about age, site of first cancer, site of second cancer, histology, grade, history of cancer in the first degree relatives, tobacco habits (smoking or smokeless), alcohol intake (regular, occasional or never) and radiotherapy for the first cancer were noted both from patient interviews and medical records. Detailed and exact quantification of exposure to tobacco and alcohol was not available in all cases. Neither were details about reproductive and sexual history available.

### DNA extraction

Having obtained an informed consent, 5–10 ml of peripheral blood sample was collected in sterile EDTA tubes. The DNA was extracted from the leukocyte pellet obtained from the blood by sucrose lysis, purified by phenol: chloroform and suspended in Tris-EDTA buffer and stored at -20°c.

### PCR

Multiplex Polymerase Chain reaction (PCR) was performed to simultaneously genotype *GSTM1 *or *GSTT1 *gene and the Human Fibroblast *Interferonβ-1 *gene (*IFNβ-1*). The *IFNβ-1 *gene was used as an internal control for amplification failure. The primer sequences were as follows

1) *GSTM1*: F: 5' CTG GAT TGT AGC AGA TCA TGC 3' and R: 5' CTG CCC TAC TTG ATT GAT GGG 3' which generates a 273 bp fragment;

2) *GSTT1*: F: 5' TTC CTT ACT GGT CCT CAC ATC TC 3' and R: 5' TCA CCG GAT CAT GGC CAG CA 3' which generates a 459 bp fragment and

3) *IFNβ-1*: F: 5' GGC ACA ACA GGT AGT AGG CG 3' and R: 5' GCC ACA GGA GCT TCT GAC AC 3' which generates a 170 bp fragment.

The *GSTM1 *or *GSTT1 *gene and *Interferon *gene were co-amplified in a 15 μl reaction mixture containing a total of 50 ng of genomic DNA as the template, 2.6 μM of each *GSTM1 *primer, or 2.6 μM of each *GSTT1 *primer, and 2.6 μM of each Interferon primer, 1.2 mM each dNTP, 1 × PCR buffer (which contained 20 mM Tris, pH 8.4, 50 mM KCl), 2 mM MgCl_2 _and 0.6 units of Taq polymerase (Amersham). The PCR profile for amplification of *GSTM1 *gene consisted of an initial melting step at 94°C for 9 min followed by 30 cycles at 94°C for 1 min, 55°C for 1 min, and 72°C for 1 min, with a final step at 72°C for 10 min for elongation. The PCR profile for amplification of *GSTT1 *gene consisted of an initial melting step at 95°C for 9 min followed by 30 cycles at 94°C for 1 min, 66°C for 1 min, and 72°C for 1 min, with a final step at 72°C for 10 min for elongation.

### Gel electrophoresis

The PCR products were separated either on 2% agarose gels or on 12% polyacrylamide gels and were seen by ethidium bromide staining or silver staining respectively. In the PCR reaction presence of a 170 bp (*IFNβ-1*) band alone and absence of a higher 273 bp (*GSTM1*) or 459 bp (*GSTT1*) band indicated null genotype of the sample. In the PCR assay when both Interferon and *GST *fragments were absent then it indicated that either the PCR was not successful or the DNA was degraded.

### Statistical analysis

Fishers exact test was used to assess the statistical significance for the difference between proportions of various factors (number of tobacco chewers, *GSTM1 *null, *GSTT1 *null, either *GSTM1*/*T1 *null).

## Results

### Overall data of all females

Forty-nine females with a paired SCC in the UADT in association with another cancer (at genital and non-genital related sites) were identified. The median time to development of the paired cancer (or second cancer) was 4 years. The median age at diagnosis of first cancer in all females was 51 years and that of second cancer was 60 years. The commonest sites of first cancer were oral cavity (n = 17), genital region (n = 15), and esophagus including post-cricoid region (n = 12). Information on family history of cancer was available in 42 women and 6 (14.3%) had a first-degree relative affected with cancer. Of the 45 women where details of habits were available, 25 (56%) were tobacco chewers whereas only 1 (2%) smoked. Of the 29 patients in whom GSTM1 and T1 genotyping was done, the frequencies of *GSTM1 *null, *GSTT1 *null and either *GSTM1/T1 *null were 35%, 17% and 45% respectively.

### Groupwise analysis

Results were further analyzed by dividing the females into two Groups (Table 1): Group 1 (n = 19), females who had a paired SCC in the UADT along with a cancer in the genital region and Group 2 (n = 30), females who had a paired SCC in the UADT along with a cancer at other sites (non-genital region). In the majority of the cases (80% in Group 1 and 86% in Group 2), UADT cancer occurred second in chronological order. The median time for the development of second cancer was 5-years (range 0–20 years) for females in Group 1 in comparison to 3-years (range 0–24 years) for females in Group 2. There were no significant differences in the median age at the diagnosis of the first cancer, family history of cancer or tobacco habits between the two groups. Uterine cervix was the commonest genital site of cancer in females in Group 1 (16/19, 84%), followed by vagina in one female, vaginal vault in one and vulva in one. The sites of second UADT cancer in females in Group 1 were: oral cavity (n = 7), oropharynx (n = 2), hypo pharynx (n = 1), trachea and Lung (n = 4), esophagus including post-cricoid (n = 4), thyroid (n = 1). In the females in Group 2, UADT was the commonest non-genital site of cancer (22/30, 73%), followed by breast (n = 6), duodenum (n = 1), and chronic lymphatic leukemia (n = 1). Histology of the cancer in the genital region in females in Group 1 was confirmed as SCC in the majority (18/19). In the remaining female the histology could not be confirmed as the records were missing, however it was confirmed that her cervical cancer was treated with radiotherapy, a treatment that is never given without histological confirmation at our hospital.

**Table 1:** Comparison of host, environment and treatment related parameters in the two Groups of females with second cancer in the UADT.

	Group 1 [n = 19]	Group 2 [n = 30]	p value
Median age at the diagnosis of first cancer [years]	50	53	0.58
Median time to development of second cancer in years [range]	5 [range 0–20 years]	3 [range 0–24 years]	0.31
Family history of cancer	3/18	3/24	1.00
*GSTM1 *null	7/13 [54%]	4/18 [22%]	0.12
*GSTT1 *null	4/12 [33%]	1/17 [6%]	0.13
Either *GSTM1*/*T1 *null	9/12 [75%]	4/17 [24%]	0.01
*Tobacco habits	9/18 [50%]	17/27 [63%]	0.54

* Except one smoker, all other habitués were tobacco chewers and none of them consumed alcohol.

Null genotype frequencies for *GSTT1*, *GSTM1 *and either *T1 *or *M1 *were more frequent in Group 1 as compared to Group 2. The proportion of females who were null for either *GSTM1*/*T1 *were found to be significantly higher in the Group 1 than in Group 2.

## Discussion

This is the first study evaluating the association of GST genotype with paired occurrence of an UADT cancer and genital site cancer. Of the 49 women with paired occurrence of an UADT SCC with a second cancer at any site, 19 (39%) were in the HPV related genital site. Majority (84%) of the genital cancers were in the Uterine Cervix and in more than half (68%) the genital cancer occurred before or within 6 months of development of the UADT cancer. Association between malignancies of the UADT and genital region (especially uterine cervix) has been observed by various investigators [13,15]. Hemminki *et al *[14] studied the pattern of second cancers in females and found a consistent increase in second HPV related cancer when the first cancer was at an HPV related site (anogenital, skin, head and neck, oesophageal and rectal cancers). An excess of oral cancers (including lip, mouth, tongue, larynx and pharynx), as found in this study, followed cervical cancers. Other investigators have also found similar results in the past [12]. Using incidence data from Surveillance, Epidemiology & End Results (SEER), Spitz *et al *[15] found an elevated Standardized Incidence Ratio (SIR) for cervical cancer after an initial buccal cavity cancer and larynx cancer.

HPV transmission has been suggested to account in part for the paired occurrence of cancers at these anatomically distinct sites of UADT and cervix and smoking has been suggested as synergistic cofactor [15]. However, smokeless tobacco is a predominant form of tobacco consumed by females in India [21] and a recent study from India has shown a positive correlation of smokeless tobacco habits with cervical cancer risk [22]. Additionally, results from another study from India found more than 80% of oral cancers in females attributable to smokeless tobacco habit as compared to the negligible influence of smoking and drinking on oral cancer in females [23]. In concordance with these figures, 44% of females in Group 1 in our study had smokeless tobacco habits and none of the patients, except for one, smoked.

The role of *GST *polymorphisms in the development of second cancers in the UADT has been a focus of few studies in various ethnic groups [4-7]. In all of these studies the *GST *null genotype (*GSTM1 *and or *GSTT1*) has been seen with increased frequency in patients with second cancers in the UADT as compared to those with single cancers in the UADT. The results of the recently published meta and pooled analysis support the notion of a greater risk of head and neck cancer when genotypes at multiple *GST *loci are considered [3]. The 35% *GSTM1 *null genotype frequency (in entire population of females with second cancers in the UADT) in this study is very similar to the *GSTM1 *null genotype frequency of 36% found in our previous study in male patients with second cancers in the UADT from similar ethnic background [7]. However, the null genotype frequency for *GSTT1 *in males was 41% vs. 17% in females from this study. Differences in the site of cancer and the form of tobacco use, which predisposes to cancer development at a particular site, between males and females might possibly explain the gender discordance in *GSTT1 *null genotype frequencies. Nonetheless the 41% and 17% null genotype frequency for *GSTT1 *in males and females respectively are higher than 8–12% *GSTT1 *null genotype frequency found in healthy controls from similar ethnicity suggesting a true association [24,25].

*GSTM1 *and *GSTT1 *null genotype frequencies (54% and 33%) in the females who had second cancer in the UADT in association with a cancer in the genital region (Group 1) in the present study are higher than a) the respective frequencies (22% & 6%) in females with second cancer in the UADT in association with a cancer at non-genital region (Group 2), b) the respective frequencies (49% & 18%) found in subjects with single UADT cancers in two studies from a similar ethnic background [24,25], c) the respective frequencies (26% & 19%) found in males with single UADT cancers from our previous study [7], and d) the respective frequencies (33% & 8% and 24% & 12%) found in Indian healthy controls [24,25]. This suggests that the females who developed second cancers in the UADT in association with cancer at genital sites (Group 1 in this study) are possibly more susceptible to damage induced by carcinogens.

The association of *GST *polymorphism and the risk of single primary cervical SCC have been studied in female patients from various ethnicities. *GSTM1 *null genotype has been found to incur an increased risk for primary cervical cancer in a mixed Caucasian, Hispanic and African-American patient population as compared to controls [9]. Like wise, in a study on 181 Korean cervical carcinoma patients, Kim *et al *[8], showed a positive correlation between *GST *genotypes and cervical carcinoma risk as compared to controls. In this study by Kim *et al*, *GSTT1 *null genotype was associated with an increased risk for cervical cancer. When the patients were stratified according to age, either *GSTM1*/*T1 *null genotype was significantly over represented in patients with cervical carcinoma who were = 40 years old. However, in a study on 190 Caucasian women, Chen *et al *[26], determined no association of *GSTM1 *null genotype with invasive cervical cancer risk. Other investigators have found results similar to that found in the study by Chen *et al *[26-28]. Differences in *GST *genotype frequencies in various control populations could account for the differences seen in various studies. For instance, the prevalence of *GSTM1 *null genotype in Caucasian, American and Korean population is similar (as high as 50%) whereas the prevalence of *GSTT1 *null genotypes varies from 15–31% in the Caucasians to 50–58% in the Koreans and Chinese.

In view of the known differences of the population frequency of null genotypes and its associated risks for cervical cancer across various ethnic groups, it is important to compare our results with those from studies conducted on subjects from a similar ethnic background. *GSTM1 *and *GSTT1 *null genotype frequencies (54% and 33% respectively) in the females who had at least one of the cancers in the genital region (Group 1) in the present study were similar to the respective frequencies (57% and 20% respectively) found in females with single (unpaired) cervical cancer cases from India [29] suggesting that the association between *GST *genotype and cervical cancer risk in Group 1 females in our study is more likely to be a true association.

HPV is undoubtedly the main causative agent in cervical SCC's and the prevalence of HPV, mainly HPV16/18 of more than 95%, has been seen in tumours from cervical cancer patients from India [30]. Evidence linking HPV to oral carcinogenesis is also growing and one of the highest prevalence rates of 74% of HPV 16 and 18 in oral tumors from smokeless tobacco habitués have been reported from southern India [31]. As HPV infections are transient, the absence of HPV DNA does not rule out previous exposure [32]. Interestingly, though most of the patients with cervical cancer are positive for HPV [17], not all patients who have HPV infection will go on to develop cancer [33]. Interactions between HPV and *GST *genotype may be a reason for this. The evidence for this is two-way. Chen *et al *[19] evaluating the reason for the lack of association between *GSTM1 *null genotypes and cervical cancer risk in their epidemiological study demonstrated that viral infection could have potential effect on the activity of xenobiotic metabolizing enzymes, particularly *GSTM1*. The results of their study raised a question of how chronic viral infections could affect cellular defenses against carcinogens in general. Evidence from other studies suggests that inherited susceptibility in the form of *GST *genotype may modulate the risk of developing HPV related cancer as evidenced by *GSTM1 *null genotype which, in addition to HPV infection and smoking, has been found to increase the risk of developing cervical cancer [9]. Moreover, in the patients with cervix cancers having a positive association with *GST *null genotype, as studied by Kim *et al *[8], HPV 16 or 18 infections were confirmed in all.

## Conclusion

The findings of our study suggest an association between the *GST *null genotype and the occurrence of paired cancers in the UADT and HPV related genital sites in females. Though this is the first study to report such an association, further confirmation from larger studies on patients from different ethnic backgrounds is required.
